# EnCTN: an enhanced AI-enabled deep learning framework for security enhancement in blockchain transactions

**DOI:** 10.1038/s41598-025-29160-6

**Published:** 2025-11-27

**Authors:** P. Bhuvaneshwari, A. Krishnaveni, Y. Harold Robinson, E. Golden Julie

**Affiliations:** 1 School of Computer Engineering, Manipal Institute of Technology Bengaluru, Manipal Academy of Higher Education, Manipal, India; 2 Department of Mechanical Engineering, Government College of Engineering, Tirunelveli, India; 3 Department of Computer Science and Engineering, Francis Xavier Engineering College, Tirunelveli, India; 4 Department of Computer Science and Engineering, Anna University Regional Campus, Tirunelveli, India

**Keywords:** Temporal convolution network, Auto encoder, Blockchain, Security, Smart transactions, Anomaly detection, Deep learning, Engineering, Mathematics and computing

## Abstract

The deep learning technique has emerged as an exemplary model for managing the Artificial Intelligence-based Blockchain framework with technological enhancements to guarantee reliable data through the consensus procedure. The deep learning-enabled blockchain transaction model has involved the development of security to solve the problems of confidentiality and data anonymity. The Hybrid techniques of the Blockchain with the Deep Learning technique are proposed to generate enhanced data durability and its propagation through the enhanced convolutional temporal network (EnCTN) for transaction analysis in a blockchain-enabled Auto Encoder technique. The sliding window extraction technique is used to extract information from a particular window size to evaluate the needed input values from the temporal series. The dilated Convolution is used to capture the long-range dependencies. The proposed technique is implemented in the Ethereum environment using Python, and experimental results show that it has produced an improved performance than the relevant technique in several performance parameters. The anomaly classification accuracy is improved than the relevant technique and it is evaluated using the NSL-KDD dataset. The proposed framework delivers an efficient solution for the real-world anomaly detection application while accurate discovery of temporal anomalies and computational efficiency is enhanced.

## Introduction

Blockchain is a decentralised technique that distributes the ledger for managing secure transactions among untrusted nodes, where the Blockchain enables the central nodes to validate the data blocks in the ledger through specific rules. The stored blocks are secured, reliable, and trusted. As the needed validation process is implemented into the network, the industry could benefit from blockchain-based solutions for the supply chain system by implementing blockchain-based code for performing the decentralised process^1^. The trust among several stakeholders in the supply chain system is crucial for providing security and traceability within the infrastructure-based system. This system should integrate devices with the mobile system, and smart contracts should utilise a specific solution to ensure shared ledger management^2^. The blockchain transaction has several real-world applications, such as supply chain management, smart contracts, healthcare, intellectual property, land registry, pharmaceuticals, cybersecurity, voting systems, and identity verification. Every stakeholder could participate effectively in tracing the products into the supply chain to ensure the identification and validation for managing the smooth process. The permission-based blockchain process is used to increase data accessibility, ensuring the supply management process for regulating data communication and implementing specific solutions to several policy-based management processes. The private blockchain is minimised to the identified stakeholders, which could achieve confidentiality and privacy, and restrict the supply chain^3^.

The blockchain process could eliminate the gap within the network system for producing traceability records. The smart contract^4^ has a shared database, which deviates from the conventional database, given that data storage is stored in blocks through the cryptography functionality, and various types of data storage are available in blockchain ledgers. It is in a decentralised format so that nobody can control the retention process for all users. The stored data is immutable and irreversible, and viewable to every user. A transaction^5^ manages the activity details that have a blockchain form. The Ethereum blockchain can transfer cryptocurrency between users, including smart contract deployment and transactions.

The decentralised framework^6^ is secured, which enables data management as edge computing permits real-time data management. The cooperative mixture of technologies could change healthcare applications by improving efficiency, minimising costs, and enhancing patient attention. Blockchain technique^7^ is a secure distributed ledger using networks that ensure data integrity. In a decentralised framework, the interconnection among the members makes the versatile tool a versatile tool in several sectors to deliver data processing. Blockchain performance in transactions exceeds the related techniques of cloud-enabled services. Security is enhanced using the flexibility in the face of hacking, even though well-known attackers could fail.

Additionally, the accuracy of the blockchain transaction through the Smart Contracts could reduce the risk that the decentralised management of blockchain minimises the resilience, as interference with the substantial advancements over the centralised cloud-enabled database^8^. Blockchain applications in healthcare units overcome exceptional challenges by providing security, complex performance metrics like latency and computational cost, and maintaining transaction security. User authentication^9^ involves the valid user in the blockchain framework executing the transaction with a higher level of protection and privacy using Blockchain technology, as the Blockchain model integrates the Deep Learning model^10^ and incorporates smart contracts to maintain secure transactions.

Anomaly detection^11^ is required to identify similar patterns using the Deep Learning framework to analyse the data. Blockchain-enabled innovative applications comprise an effective procedure for processing large volumes of data. Deep Learning techniques enable the machines to learn without the direct coding concept; the primary function is to process the input to identify statistical patterns. The Blockchain benefits from enhancing the incentive-enabled practical framework for permitting the clients to support the proposed model by eliminating the error data for improved efficiency. The Deep Learning framework is used to enhance a particular device at no cost. The efficient client data input has been intermittently measured and will be accessible to users. The main contribution of the paper is:

- The distributed deep learning framework is developed to maintain anonymity through cryptographic functionalities.
- The sliding window extraction model is used for extracting the data from the beginning state, while the extracted frame is used to measure the accumulation of frequent patterns.
- The Convolutional Temporal Auto Encoder technique is constructed with a deep learning model to complete the anomaly detection in the time series.
- The proposed EnCTN model is incorporated and evaluated for efficiency through the performance metrics of throughput, Processing time, and power utilisation.

## Related works

A blockchain-enabled architecture is used effectively in secure transactions to minimise the complexity of the multi-chain functionality. The zero-knowledge framework^12^ is used to prevent information interconnections and keep access to the unconfirmed personality, and the flexible computing framework with the monitoring model enhances big data management. The blockchain-enabled data storage model^13^ is associated with the blockchain and big data frameworks for accessing valuable data from IoT-based components to efficiently solve the problems of big data and maintain the confidentiality of the patient. The computational overhead, complexity, and privacy requirements necessitate the development of a solution for healthcare monitoring. The blockchain model enables patients to share health information effectively, and smart contracts are used to manage authorised users updating patient records. The novel healthcare model incorporating the blockchain uses the hybrid technique^14^ for optimising the key values. The symmetric and asymmetric encryption are combined to minimise the complexity. A vast range of healthcare-related issues has been solved using Reinforcement Learning^15^ with Blockchain technology as the framework, which has several deployments of Machine learning approaches for providing the solution to application-related problems. Specifically, the architecture is limited through various resource optimisation methodologies capable of accommodating the system-oriented framework, which includes the time, resource utilisation, and offloading of blocks.

The deep learning framework^16^ could not provide privacy in the training state where the training information is saved autonomously in blockchain as the transitional gradients are saved in the standard storage of the server to utilize the significant information for the training data, the security method with the data gradients for data uploading to achieve information security through the training accuracy. The GAN learning process^17^ provides privacy in the intermediate server to conduct the link capability attack on intermediate gradients, and it provides sufficient data features. The spatial domain securely encodes data with the service attribute, while the homomorphic encryption technique involves the collaboration of applicants.

The Ethereum smart contracts^18^ have been analysed through transaction-enabled analysis. The analysis has tracked the smart contracts through a specific dataset and generated an enhanced database using the data slicing method. The LSTM framework^19^ was used to test the dataset after performing rigorous experiments. The anomaly detection process in multi-layer networks must identify abnormal structures in the blockchain transaction process to understand the network’s structure and detect changes in the network topology. The anomaly detection process^20^ with the Ethereum blockchain model analyses phishing detection based on feature learning and transaction record enhancement. A SIEGE method^21^ is used for identifying the spoof scam on Ethereum as the primary transaction data for constructing the transaction graph on every split to a temporal task for facilitating data flow within dissimilar graph splits. Blockchain-based Secure, Interactive, and Fair Mobile Crowdsensing (BSIF) technique^22^ requests every user to verify their identity using private keys in the registration stage, as the location-enabled symmetric key generator is involved for coordinating the session key to target value identification. A fully decentralised technique, DecChain^23^, has provided the solution for the permissioned blockchain. At the same time, the authentication procedure utilises a trusted entity to enhance security within the edge servers and specific users to eliminate bottlenecks.

A novel Group Theory binary spring search technique^24^ has implemented a hybrid deep learning model to efficiently identify the intrusion in the IoT network, as it initially implements the privacy-preserving approach through the Blockchain technique. Patient health record security is the central aspect to protect from several security vulnerabilities. The homomorphic encryption procedure ensures secure access through the secure key revocation function to update several policies. A lightweight authentication procedure^25^ has been implemented to use a deep learning framework for facilitating decentralised authentication within IoT devices. The validation latency within the communication model involves enhanced communication. The Patient health record security^26^ is the primary encryption process for database access, ensuring secure data storage through homomorphic encryption. This enables users to access data in a safe manner. The Hyperledger tool is used to construct smart contracts for monitoring user behaviour. A hybrid deep learning technique^27^ has enabled the effective processing of complex healthcare data, which includes sensor data and medical records, through a permissioned blockchain model, allowing authorised users to access sensitive data through a privacy-preserving model.

The main challenge of blockchain transactions is scalability, as it is hard to process a huge number of transactions per second. The blockchain is secured against cyberattacks and other security threats related to smart contract issues. The regularity uncertainty for blockchain transactions, which involves communicating and interoperating with the challenges of the Proof of Work procedure, requires additional energy. Smart contracts have complex issues that are difficult to eliminate through formal verification and auditing, which enhances security. The smart contract-related issues will minimise the security of the Blockchain-enabled transaction process by adapting the verification and auditing process, which ensures the security of the smart contract.

The Machine Learning technique has been involved in detecting botnet attacks by delivering into the public datasets, the effective pre-processing technique enhances the feature extraction process in IoT security^29^. The efficient IoT threat detection technique^30^ has been utilized to provide the highest accuracy and the capability for adapting to various threat environments. The robust security has been ensured by leveraging an integrated blockchain technique, as the central control centre facilitates the sharing of identified threats through various classifiers^31^. The hybrid dense neural networks (DNNs) and logistic regression (LR) technique^32^ has been utilized to enhance privacy and security by including an extra protection layer.

## Proposed work

The proposed model is constructed to manage the decentralised framework for implementing blockchain transactions, which enhances privacy. The cryptographic functionalities are utilised to maintain anonymity with accessibility and privacy, while the proposed model is secured to inherit the Distributed Deep Learning model. The intelligent framework-based transaction is related to the smart contracts for initiating the transactions, where the aggregate gradient from the untrusted entities prepares the updated parameters to enable the transaction. The smarter blockchain transaction has learning features that could be encompassed by blockchain-enabled applications and protected by the distributed ledger to enhance the utilisation of Deep Learning. It could be utilised for improving the time needed to maintain consensus using enhanced routes for data exchange.

**Fig. 1:**
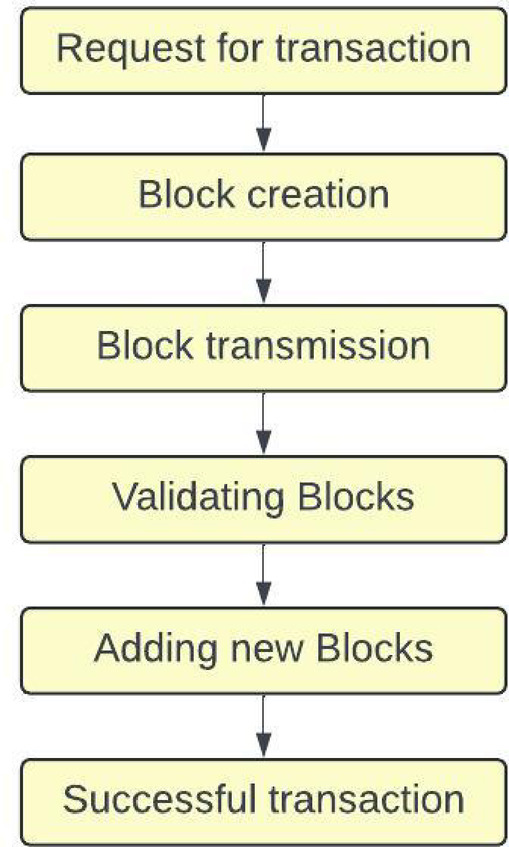
Blockchain schema.

Additionally, it achieves the proposed model to enhance the blockchain-enabled distributed model to produce innovative applications. While combining Deep Learning with Blockchain has some issues, as deep learning model training requires specific computational resources that could require more energy, scalability is another concern. The complexity of creating a smart contract can make the development and deployment process more challenging. Data privacy is affected when sharing sensitive data. The smart contracts gather data from several sources, such as IoT devices, intelligent machines, and cameras. Figure 1 demonstrates the blockchain schema for producing a successful transaction.

The blockchain serves as the primary source of coding through Deep Learning. It could be involved in data interpretation for estimating the application by removing data faults like redundancy, data loss, noise, and errors. The proposed model incorporates the features of deep learning and blockchain, which may solve the issues related to data management in Deep Learning models effectively. The transactions in the secured data transmission have carried out the notification from the financial markets to exchange the Hyperledger fabric to maintain the unique identifier with the representation of the sender, the Ether native encryption, which delivers the data to the receiver and the external layer is utilised to receive the data. Every smart contract could recognise the cryptographic steps to enhance the process execution within the transaction in Bitcoin to evade the threats to the Blockchain model. Further data is analysed while the receiver is trained to submit the query for every measurement state that could be reached in the smart contract.

**Fig. 2:**
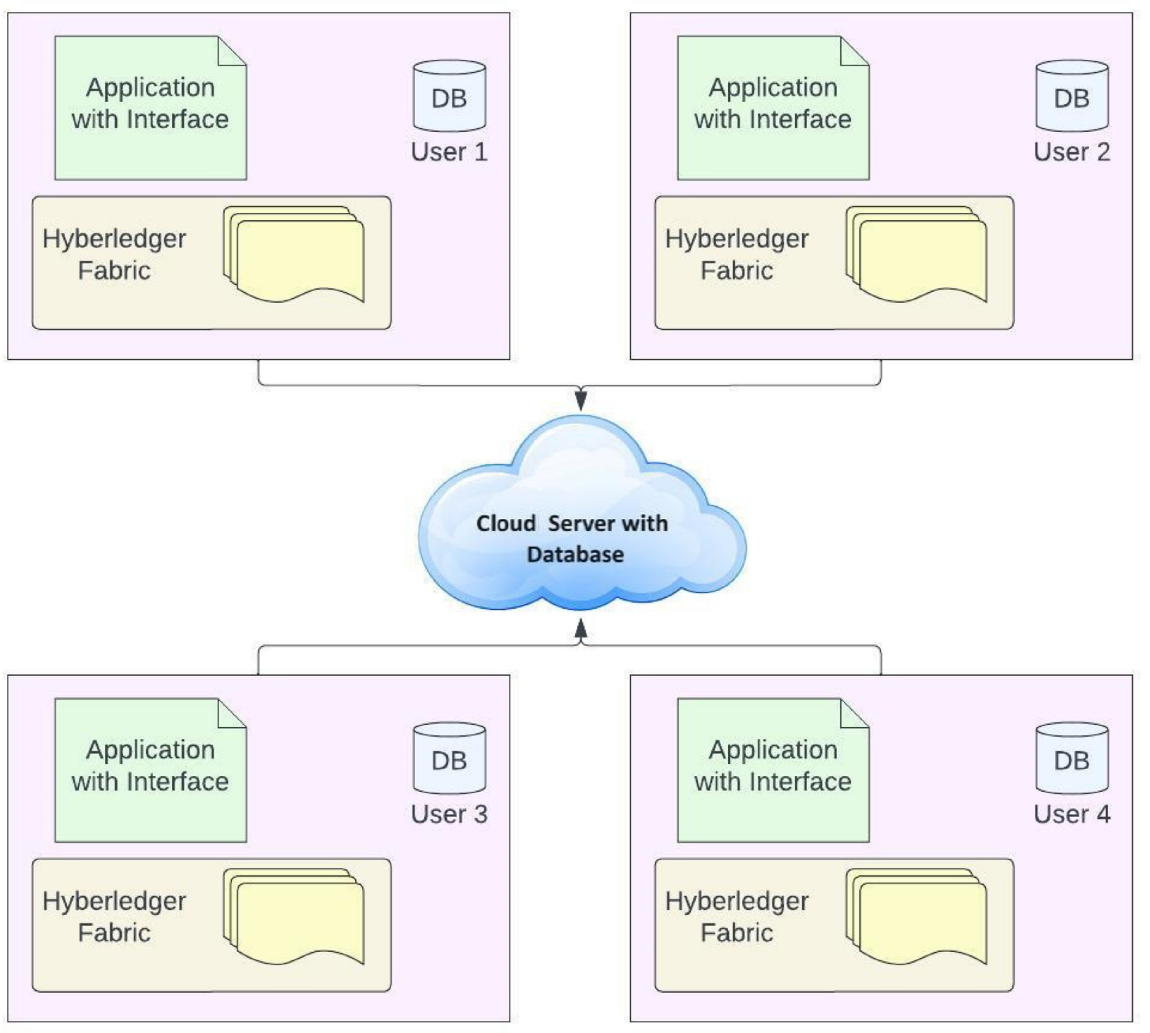
Blockchain framework for transactions.

The payment model of a single account has statistical records that collect data consecutively within a timeframe through several experiments that have measured the deviation in the extensive analysis and the utilisation of machine learning techniques to collect the observational information without including the volume and the frequency. The Resnet-enabled techniques in the supervised learning model require a massive amount of data, which needs more time. Every address transaction data has minimised the unequal recognition technique that could be framed through the RNN model for producing the regression analysis. The neural methods are used to perform several regression analyses to order the time series, which involves a feature extraction process where methodologies from the traditional intrusion identification model can be developed from the temporal information. The payment timeframe and the interest during the payment period were for every domain name, in exchange for a regression model in the temporal series. The sliding window extraction model is used to extract the data from the initial state of specific window sizes that need to be consecutively evaluated and require the number of readings in the temporal series. The extended frame has introduced the random series for measuring the accumulation of frequent patterns, where the sliding window technique is involved in a single payment. Figure 2 demonstrates the Blockchain framework for a transaction.

**Fig. 3:**
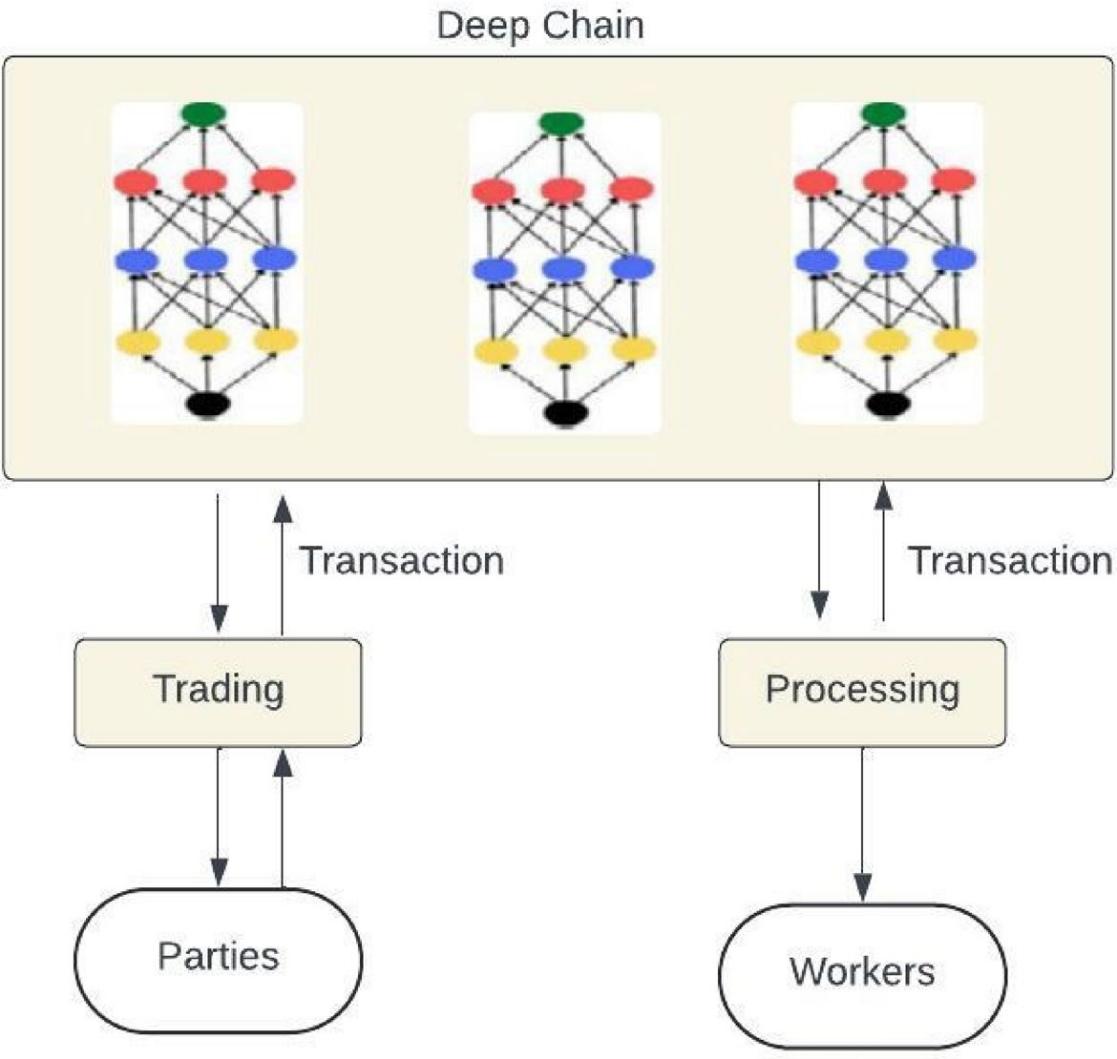
Distributed deep learning communication.

The Convolutional Temporal Auto Encoder Technique is constructed with a deep learning-enabled method to implement anomaly detection in time series data, as it deviates from the CNN model in that it combines the residual blocks and auto-prediction. Every sub-block contains a group of convolutional layers with an activation function and a dropout layer. The residual blocks are included in the output layer, where the proposed model has the filters and kernel size, and the output of every block produces the output sequences. The encoder identifies the essential features of the input sequence for producing the reconstruction in the consecutive stages to segregate the valuable features in the long-term patterns. The 1 × 1 Convolutional layer is adapted to minimise the feature map dimension, and at last, the pooling layer helps to down sample through the time series. The decoder is used for reconstructing the original sequence from the encoder output, where the length has been restored. The unsampled sequence has entered into the transaction block with a similar framework in the encoder block, and the dimension has been restored through the 1 × 1 convolution layers and the filters. The training process has focused on the compressed encoders from the input sequence, which permits the exact reconstruction process where the nominal patterns have a small number of errors in a time series. It aims to reduce the reconstruction error from the minimum data in the training process, as demonstrated in Fig. 3.

**Fig. 4:**
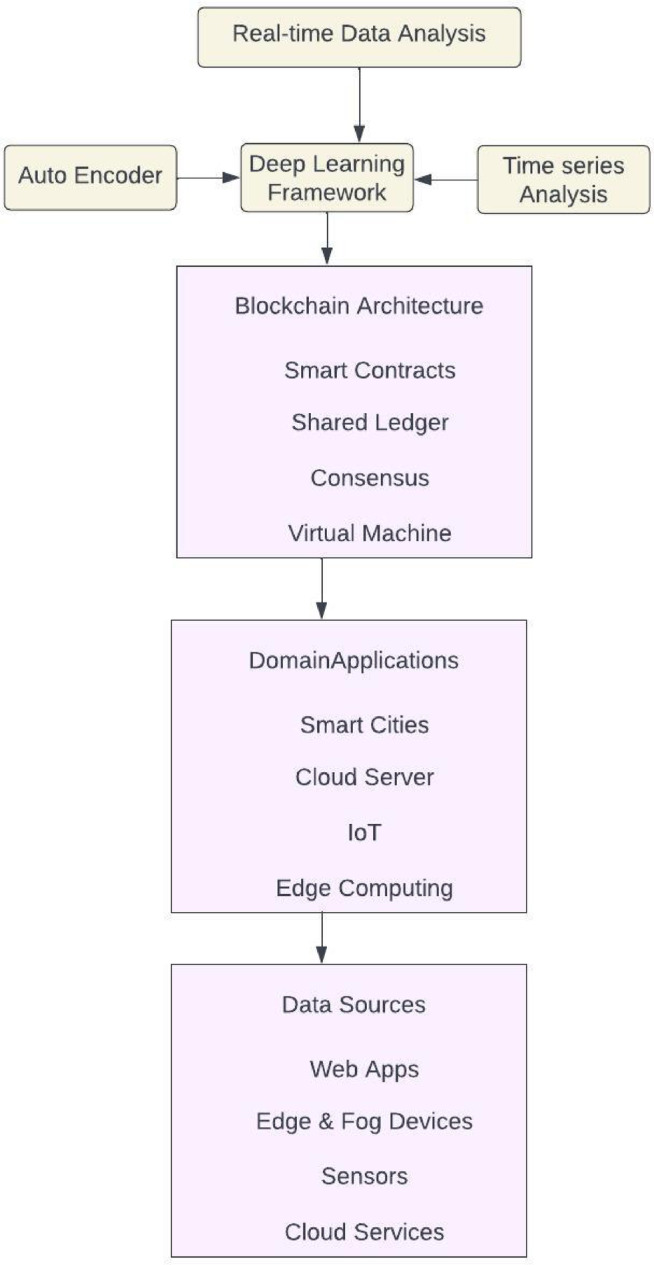
EnCTN communication process.

The abnormal behaviour is identified through the sliding window of a specific length to gather the dimensional vector into the error matrix; the sliding window is used to reduce the noisy elements. The outlier detection process has identified the anomaly points in the dimensional space. The outlier detection process provides the anomaly detection score based on the threshold value for the convolutional time series. Figure 4 demonstrates the EnCTN framework with the Deep Learning concept to implement Blockchain technology for innovative applications like IoT devices. The blockchain involves smart software that can be included in the data to estimate the application to minimise data faults like noise, data loss, and errors.

The min–max normalisation procedure is used for performing the pre-processing, where every feature’s minimum and maximum values are assigned as 0 and 1, respectively; the value is computed in Eq. (1).

1 $$P{P}_{new}=\frac{PP-P{P}_{min} }{P{P}_{max}-P{P}_{min}}$$

where PP demonstrates the feature group, $P{P}_{min}$ is the minimum value, and $P{P}_{max}$ is the maximum value. The feature selection procedure is used for identifying the features. The traditional technique of optimisation begins as the path is identified between the starting and ending points of the nodes. The initial step is to determine the search agents from the entire values; the beginning value is ${m}_{0}$, and the individual optimisation is computed using Eq. (2).

2 $$O=\left\{{O}_{1}, {O}_{2}, \dots .., {O}_{n}\right\}$$

The fitness function is computed in Eq. (3).

3 $$F{F}_{n}= \sum_{i=1}^{n}\frac{Len}{A{S}_{t}({X}_{i})}$$

where $F{F}_{n}$ is the fitness function, Len denotes the length, AS is the Average Speed, and X demonstrates the individual value. The Objective function is computed in Eq. (4).

4 $$O{F}_{i}\left(x+1\right)=\left(1-\gamma \right)O{F}_{i}\left(x\right)$$

The dynamic movement is computed using the adjacent probability values in Eq. (5).

5 $${n}_{i}\left(x+1\right)={n}_{i}\left(x\right)+S\left(\frac{nk\left(x\right)-{n}_{i}(x)}{|\left|nk\left(x\right)-{n}_{i}\left(x\right)\right||}\right)$$

The fitness value is computed in Eq. (6).

6 $$FV=\beta \left(D\right)+\alpha \frac{|C|}{|S|}$$

where $|C|$ denotes the Cardinality, $|S|$ is the entire feature summation. The identification and classification of cyber attacks have been performed through the improved proposed technique, as the neural network concept, which utilises the recurrent process to exploit the joined input values with the output values, and the pooling process is computed in Eq. (7).

7 $$\left\{f{g}_{x}, o{g}_{x}\right\}= \delta \left({M}_{fg,og }. {n}_{x}\right)$$

8 $$c{h}_{x}=\mathrm{tanh}({M}_{ch}.{n}_{x})$$

9 $$\widetilde{{ch_{x} }} = ch_{x - 1} \odot fg_{x} + \widetilde{{ch_{x} }} \odot \left( {1 - fg_{x} } \right)$$

10 $$hl_{x} = ch_{x} \odot \,og_{x}$$

The recurrent value is identified as the weighted summation for producing the speed computation. The probability value of the activation function includes the ReLu function, while the hidden layer cannot be increased further. The Hyperparameter Tuning technique identifies the hyperparameters with optimised values in regions of updated location as safer areas related to improved values of the objective function. The meta-heuristic functionality manages the group of solutions computed with Eq. (11).

11 $${A}_{i}=lv+random\, x \left(uv-lv\right), where\, i=\mathrm{1,2},\dots N.$$

where $$uv and lv$$ demonstrate the upper value and the lower value of the vector dimension, the random values lie within 0 and 1. The optimised safer location is identified through the specific position for identifying using Eq. (12).

12 $${A}_{i}\left(x+1\right)=sp\left(i\right)+\mathrm{cos}\left(2\pi \right)*\left(sp\left(i\right)-{A}_{i}\left(x\right)\right)$$

The common solution group identifies the optimised solution for computing the fitness value by searching for dissimilar states with the highest random values to determine the global area in the identifying state. The exploitation state generates the smallest random value for the exact solution. The adaptive parameters of every location are computed using Eq. (13).

13 $$\beta = Random \, \otimes \,index$$

where the index demonstrates the index value, Random has the random value, while the mean position is computed using Eq. (14).

14 $$mp=\frac{1}{x} \sum_{i=1}^{x}\overrightarrow{{A}_{i}}$$

The Euclidean distance is computed in Eq. (15).

15 $$ED= \sqrt{\sum_{i=1}^{d}{\left({A}_{i}-m{p}_{i}\right)}^{2}}$$

The agent for finding the smallest distance in the safer area has a variety of solutions. As the attack happens, the escape policy moves towards the safer space, and the optimised safer area has been identified. The fitness value is used for obtaining the solution in classification. It demonstrates the positive values for indicating the fitness and is computed through the error ratio in Eq. (16) and Eq. (17).

16 $$FV\left({a}_{i}\right)=ErrorRatio \left({a}_{i}\right)$$

17 $$ErrorRatio \left({a}_{i}\right)=\frac{Total\, misclassification}{Total\, values}*100$$

**Fig. 5:**
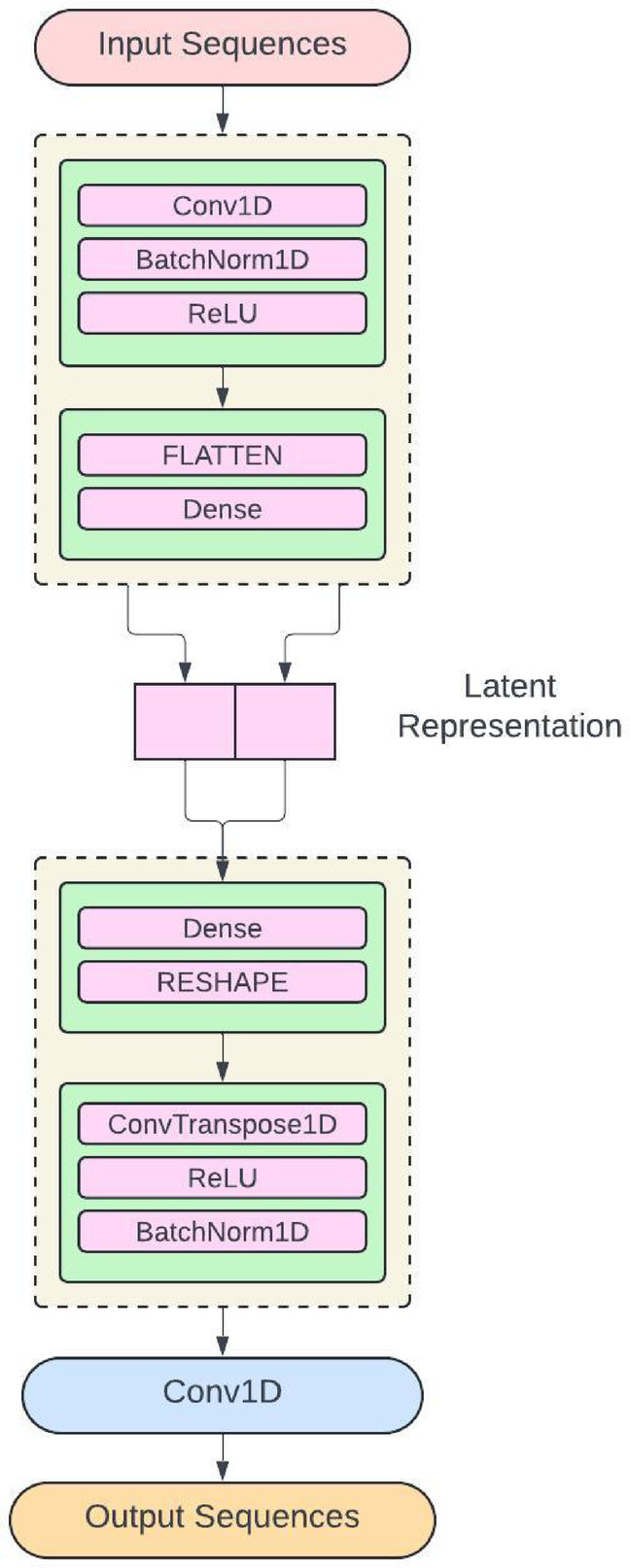
Convolutional temporal auto encoder architecture.

The time series data with the parameters of features, batch size, and the sequence length for developing the Convolutional Temporal Auto Encoder Technique utilises the dilated convolutions for capturing the long-range dependencies. The ReLU activation function is used for the Encoder process, while Convolutional transpose layers are used for the Up-sampling layers. The Hyperparameters are the total convolutional layers, kernel size, Pooling size, and total units in dense layers with the activation functions. The main advantages of this proposed algorithm are the complex temporal patterns, the identification of the noise and the missing components, and enhanced feature learning through improved detection ability, as shown in Fig. 5. Algorithm 1 shows the Convolutional Temporal Auto Encoder Technique, which has the input parameters of Threshold and Batch size. The algorithm identifies transactions within the timestamp. The input parameters are initialised through convolutional auto-encoders. The model is trained in every epoch within the batch size. The encode operation is used to find the reconstruction error and to produce the Convolutional matrix.

**Algorithm 1:**
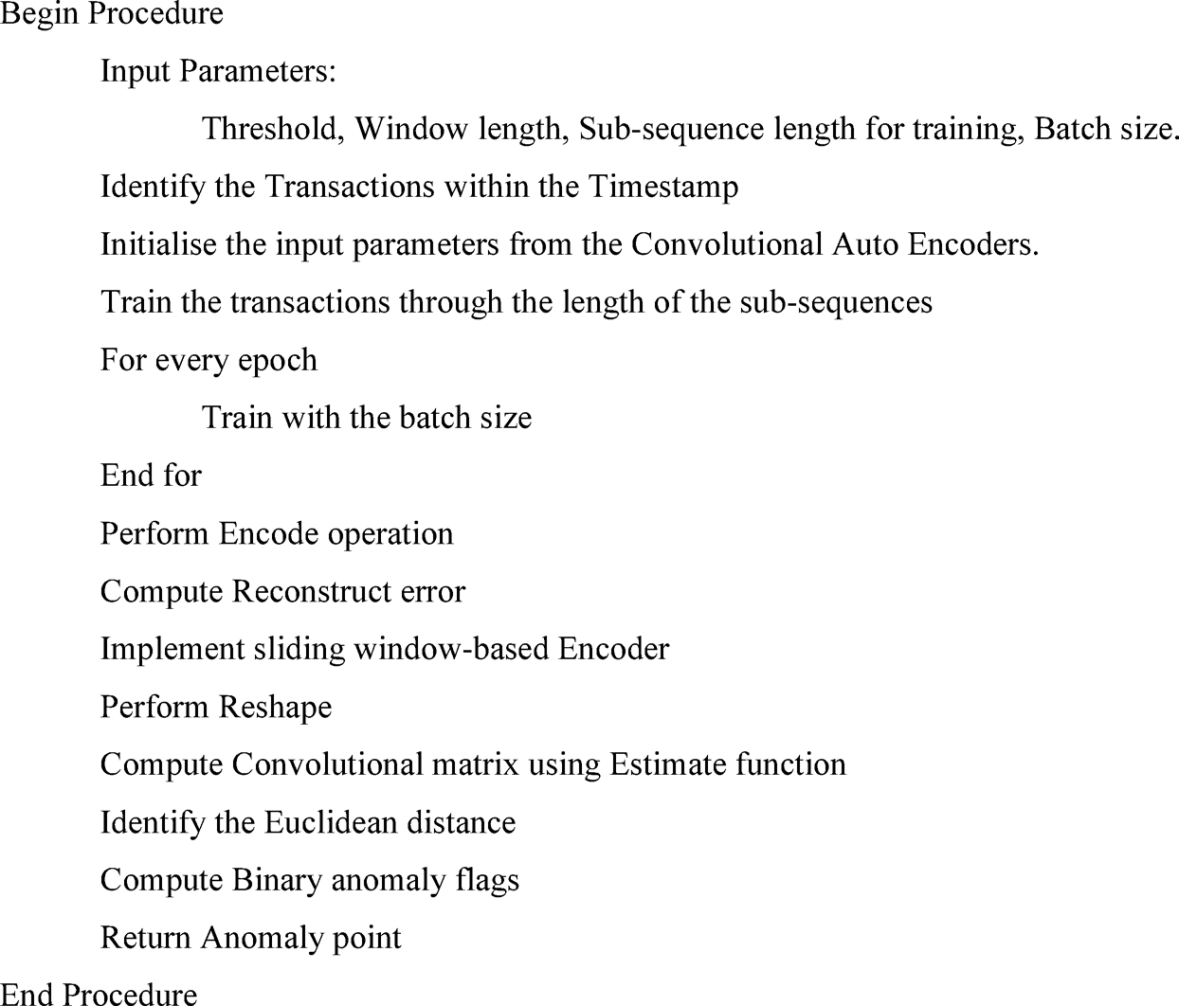
Convolutional Temporal Auto Encoder Technique

**Fig. 6:**
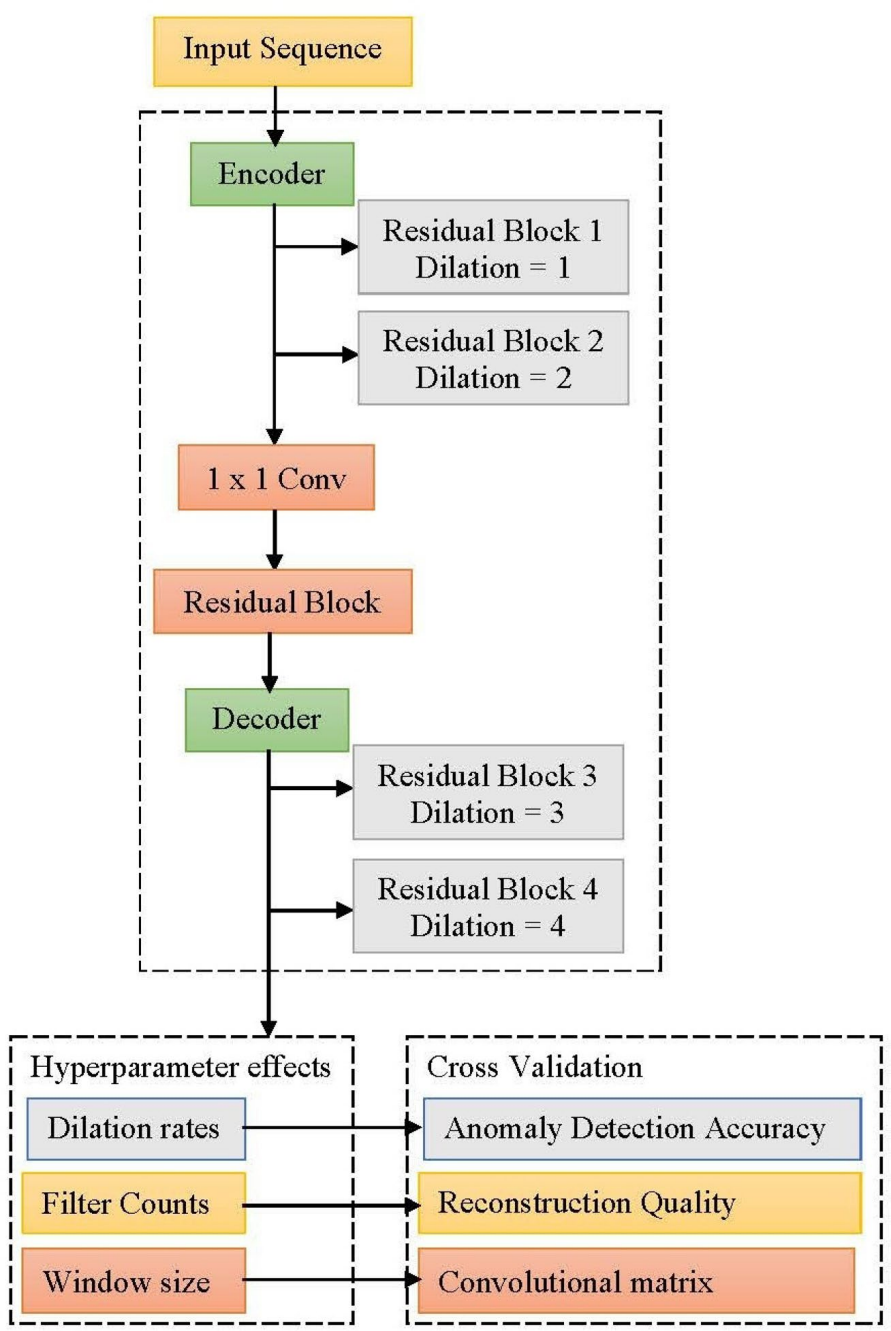
Proposed encoder-decoder model.

The proposed framework demonstrates an enhanced model to perform temporal anomaly detection by leveraging a deep convolutional framework, particularly capturing complex patterns in time series data. The framework provides an encoder-decoder model to transform the input sequences on convolutional layers into a latent representation, and the decoder produces the original input from the compressed functionality. The residual blocks have addressed the vanishing gradient problem in deep networks to enable efficient training of the traditional models. The residual connections permit gradients into the network to preserve essential feature data in several layers for enhancing the training stability. The 1 × 1 convolutions have components that contain residual blocks to transform the features for suitable reconstruction. The hyperparameters are optimized into the temporal field as the dilation rates enable capturing the dependencies for maintaining computational efficiency. The window size has to be determined accurately to capture the sequential patterns. The framework has a low variance in validation folds, which determines generalization to establish the EnCTN as a reliable solution, and the framework is illustrated in the Fig. 6.

## Performance evaluation

The proposed technique is evaluated and compared with the relevant methods in the real-time environment to identify the efficacy of the transaction offloading procedure with the Ganache tool to manipulate the transactions. The evaluation of the proposed network has the offloading procedure in the toolset, despite achieving the highest transfer speeds and minimising latency through the Ganache Ethereum. The Fedora 34 VM is running at a speed of 3.06 GHz. At the same time, the Android mobile is equipped with the latest specifications, utilising IEEE 802.11g Wi-Fi wireless transmission to connect to the edge cloud computing model through the blockchain client. The transactions were executed continuously over 24 h and randomly evaluated for 2 weeks by the transformation of the cryptocurrency in the specific transaction. The Blockchain framework is assessed through a Ganache server with several mobile users for producing data, while every timeslot has a particular task for managing the latency and the throughput. The proposed technique is compared with the relevant methods of GAN^17^, SIEGE^21^, BSIF^22^.

The power-enabled coefficient through the Ganache server as the constant voltage is assumed and evaluating the feature extraction, the proposed algorithm is involved with the gradient function, the hidden layers, and the activation function where the SoftMax activation function has the offloading decision variables and the performance of the blockchain is evaluated through the energy, delay and the execution of the transaction. The experiments are conducted for blockchain transactions in the proposed technique, which utilises 120 transactions per second. Proof of Work (PoW) is involved to provide the solution for a complex mathematical problem by validating the transactions, including the new blocks. Proof of Stake (PoS) is utilised in Ethereum, where the validators are selected to update the new block based on their stake. The key size is illustrated in Table 1.

**Table 1:** Key size details.

Key type	Size
Public key	512 bits
Private Key	256 bits
Block hash	256 bits
Signature	1024 bits
Transaction ID	512 bits
Transaction hash	256 bits

**Fig. 7:**
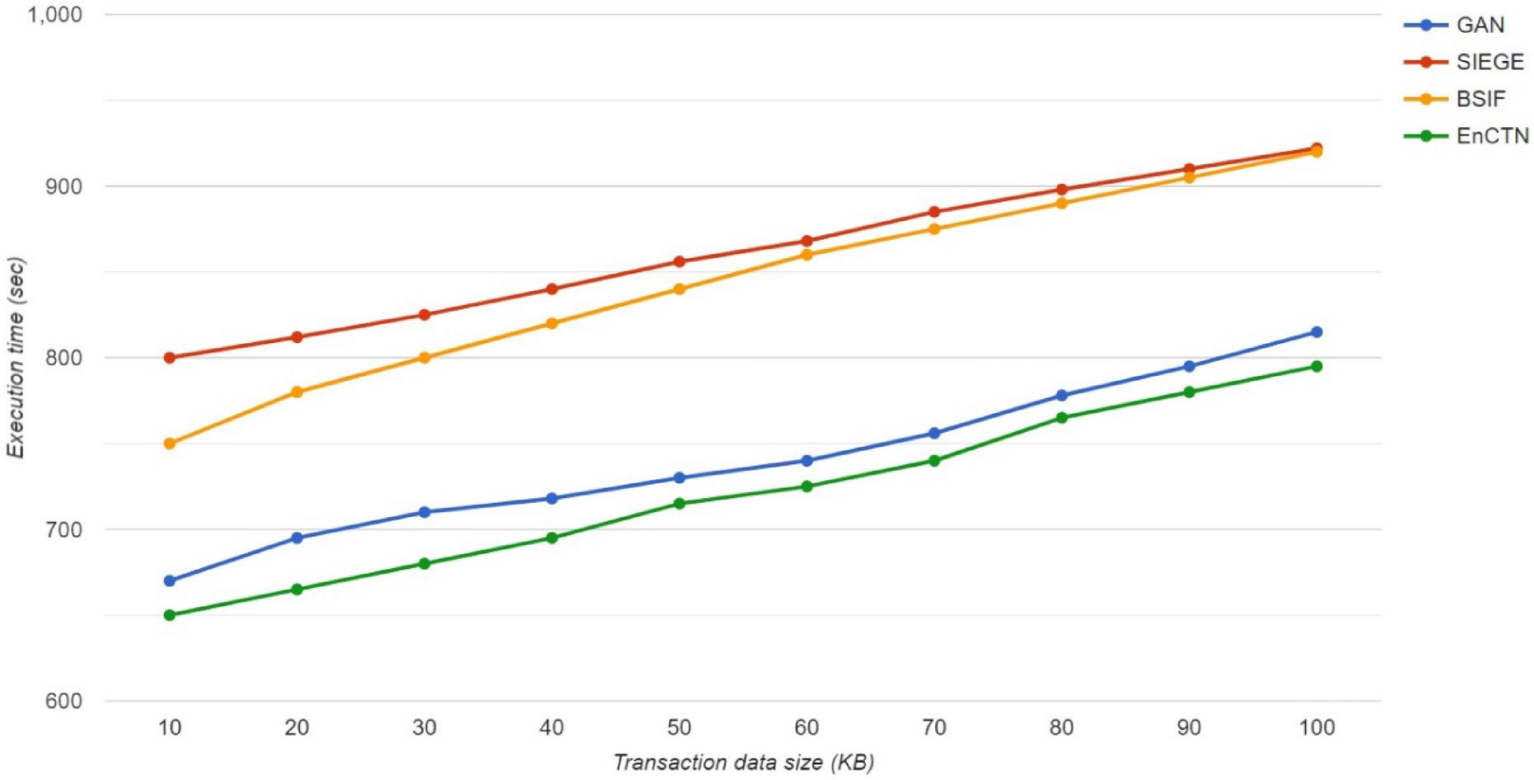
Mean processing time.

The effectiveness of the proposed model is evaluated with edge computing for executing the IoT-enabled data management while the transaction information is utilising the processing time. Figure 7 demonstrates that every blockchain transaction file has the mean processing time for computing the offloading data in the edge computing model.

**Fig. 8:**
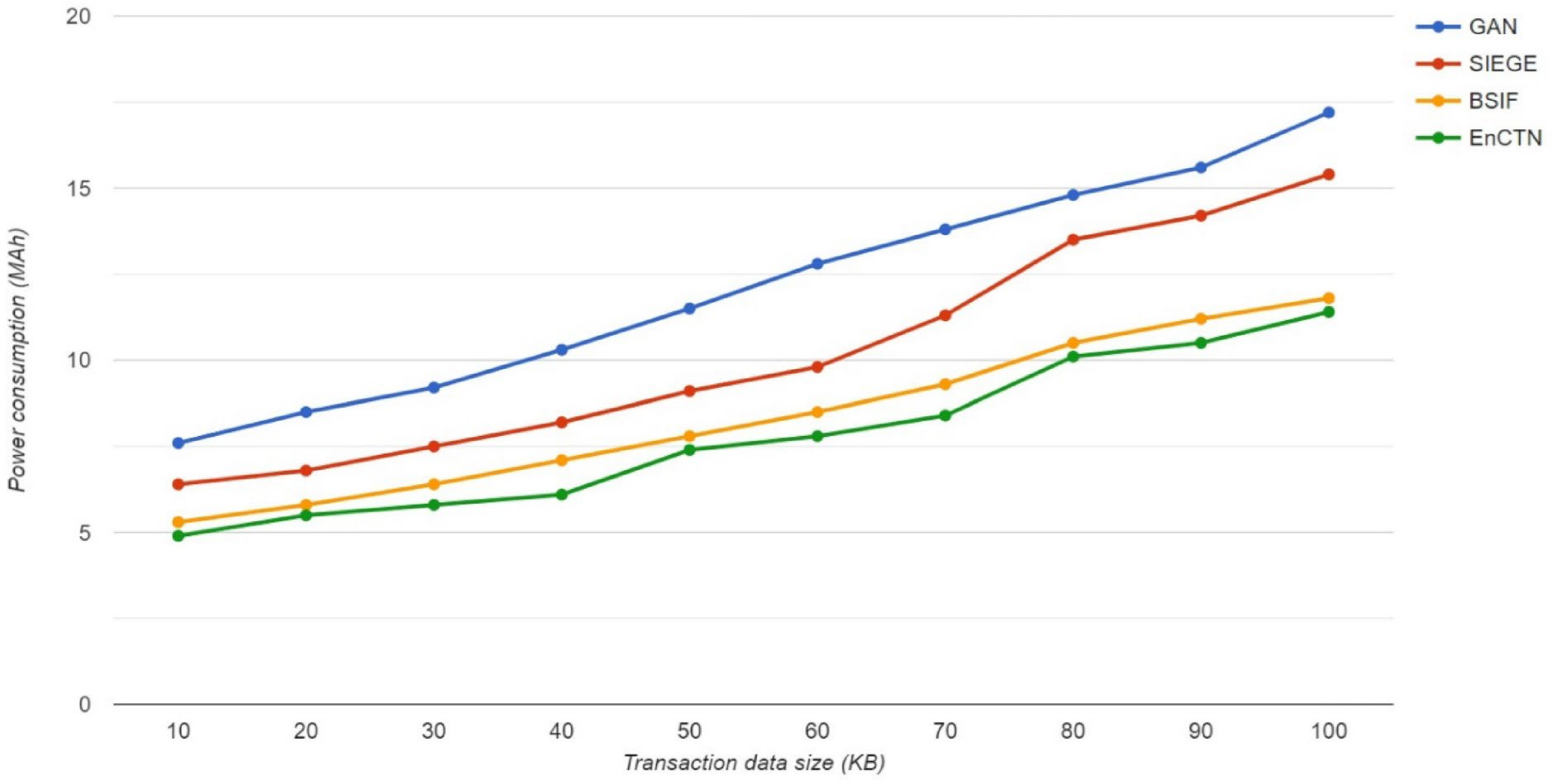
Power Utilization.

The Blockchain-enabled transaction has an offloading process; the proposed framework utilises a small amount of energy because of the resource-based computational task, and the comparison is described in Fig. 8. While uploading the data file requires 12.5% less energy during the on-demand computational model, the offloading procedure is much more energy-efficient, as the proposed transaction offloading model saves up to 17.5% more than relevant techniques.

**Fig. 9:**
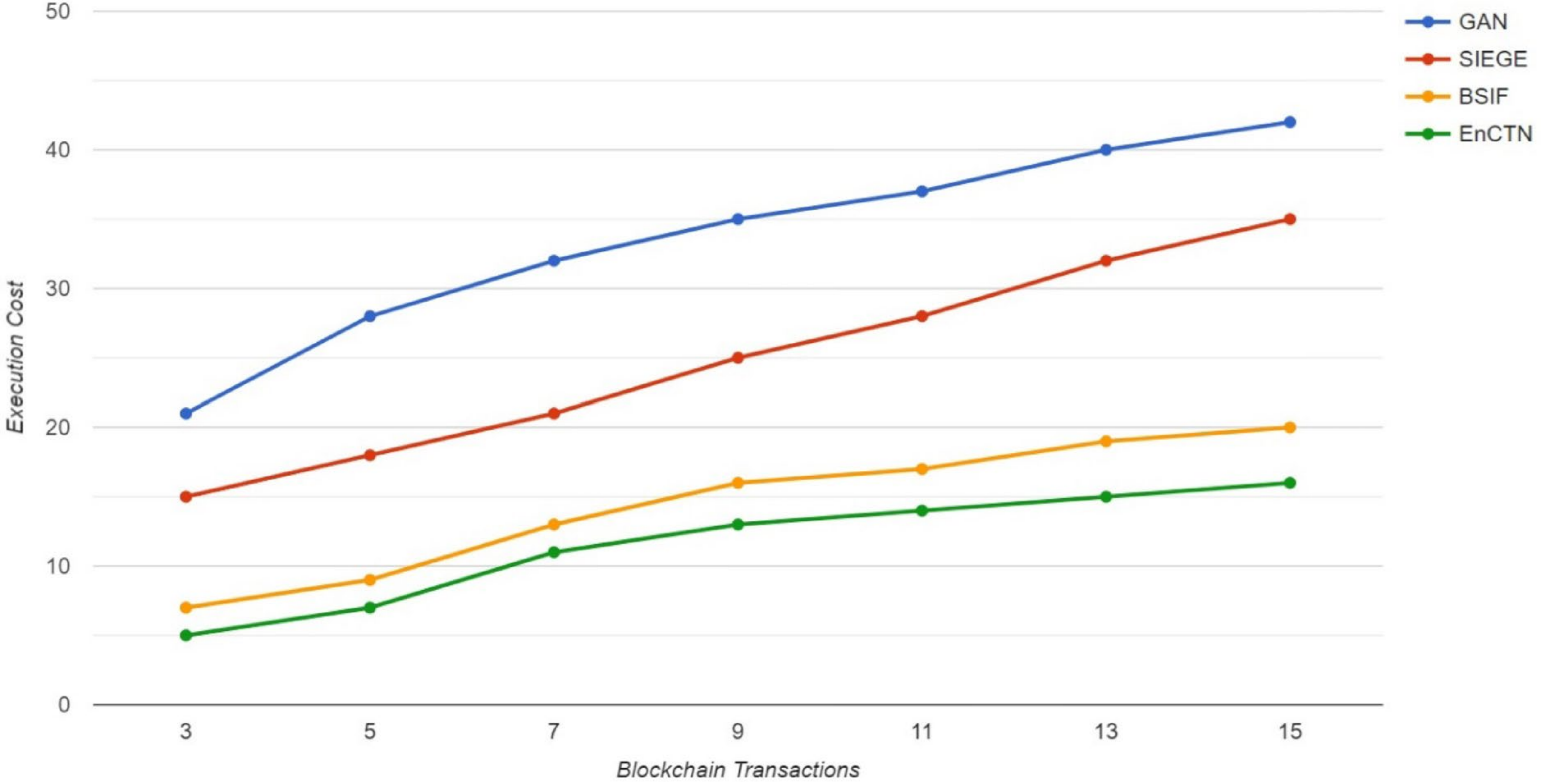
Execution cost.

The Convergence cost analysis is performed for the proposed technique compared with the relevant methods by imaging the learning factor value of 0.001 within the training process. A specific user is involved in the simulation model, while the total transactions at every value have been modified with different sizes in each blockchain transaction. The Executional cost is demonstrated in Fig. 9.

**Fig. 10:**
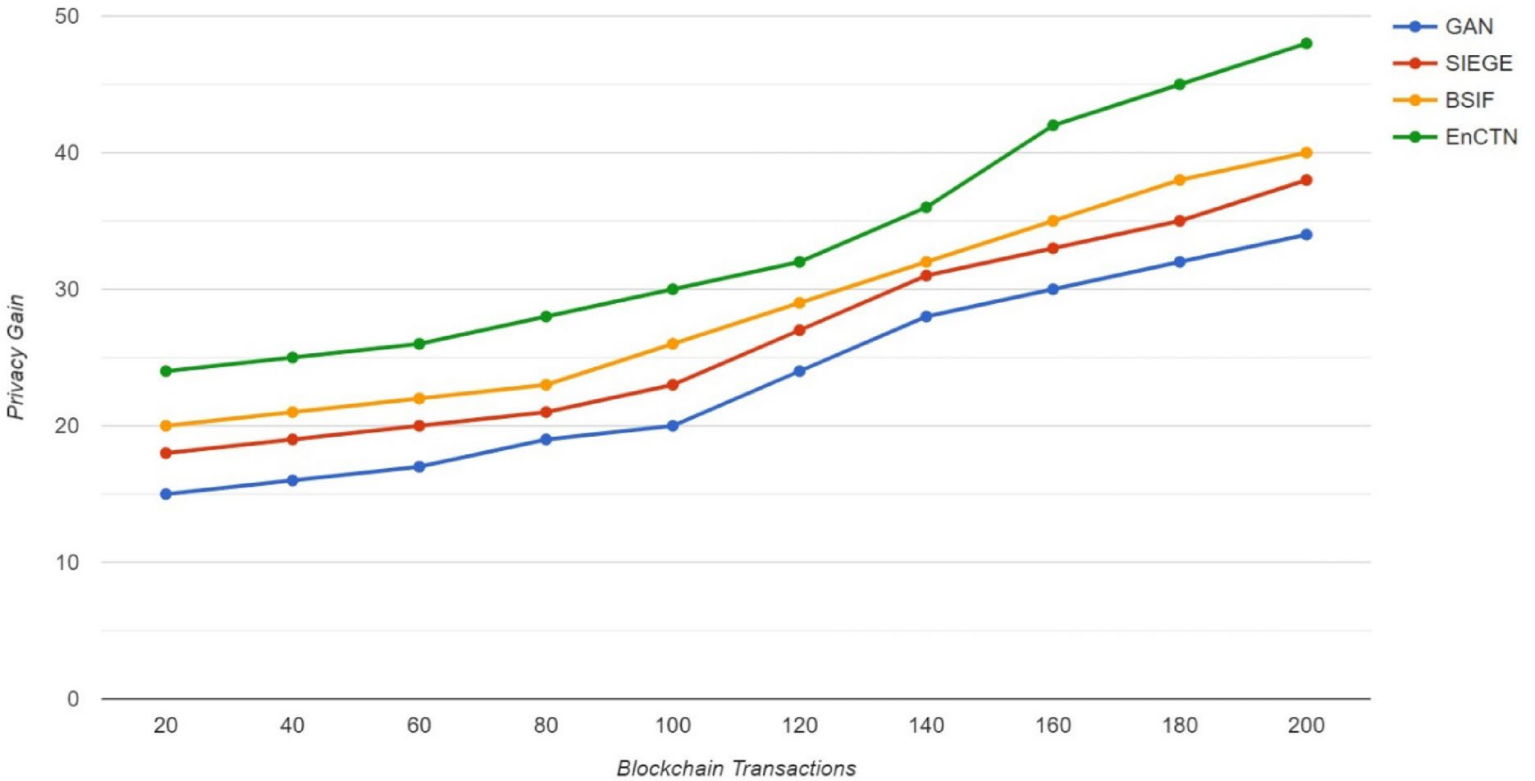
Privacy gain.

Whenever the total transaction has been enhanced, the cost of several parameters, such as the transaction data in the blockchain model, improves steadily. The rational value is identified as the data processing function increases, and the communication within the blockchain transactions should be delayed in the buffer services. The higher processing time and increased power utilisation will reduce the performance of the specific methodology, but the proposed system has been developed to overcome this decreased performance. Whenever the transaction data grows, the timestamp for every privacy level has produced better privacy management, as Fig. 10 demonstrates that the proposed technique has enhanced performance over the related methodologies.

**Fig. 11:**
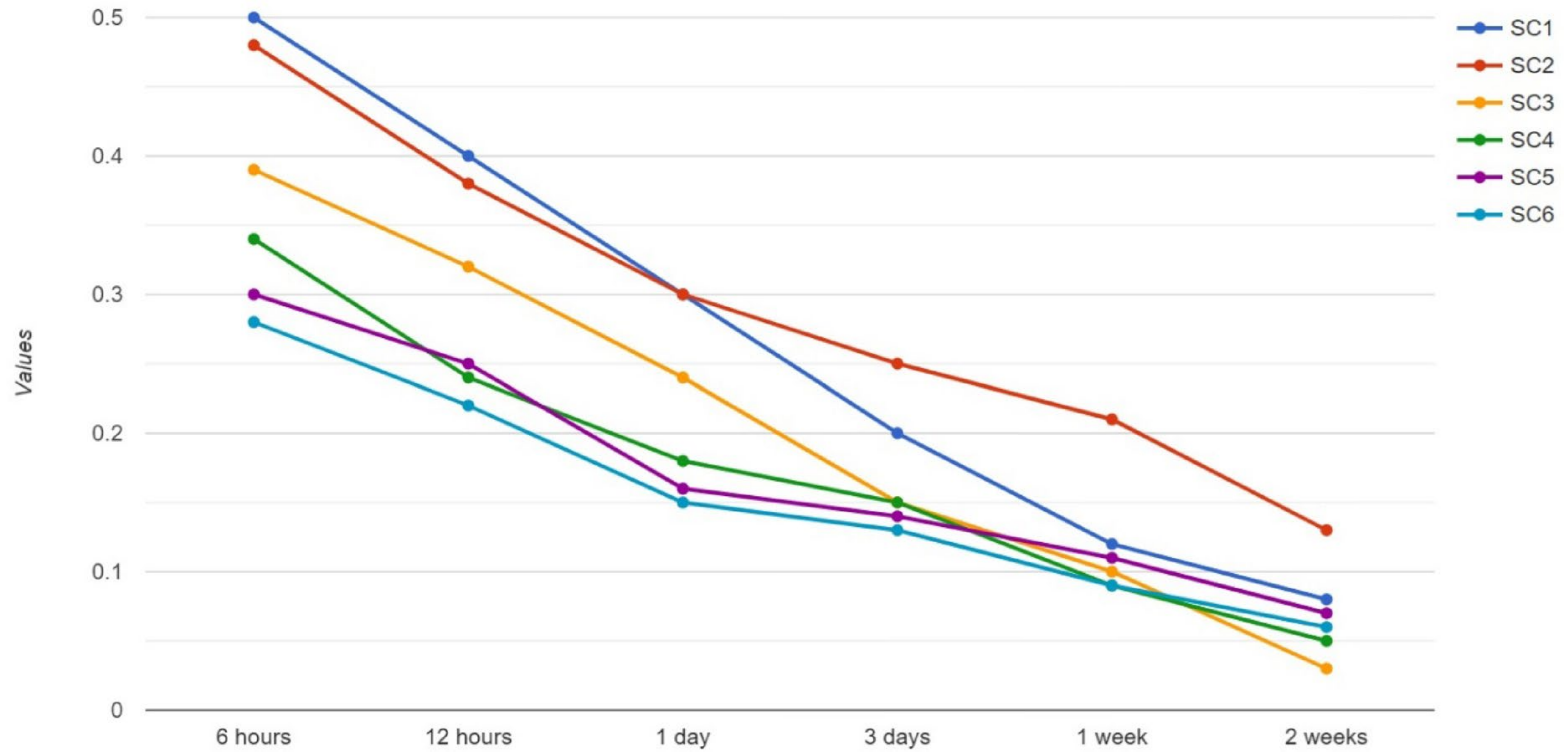
Transaction ranking.

The main parameters are evaluated by the proposed technique, which is identical to the transaction input to produce the output values. The equivalent model contains a range of 0 to 1 feature, where the highest value yields the best result. In the various aggregations on every window, the value is added to a single window to compute the specific values for every address. The extracted transaction features in several timestamps with the Smart Contracts (SC) are illustrated in Fig. 11.

**Fig. 12:**
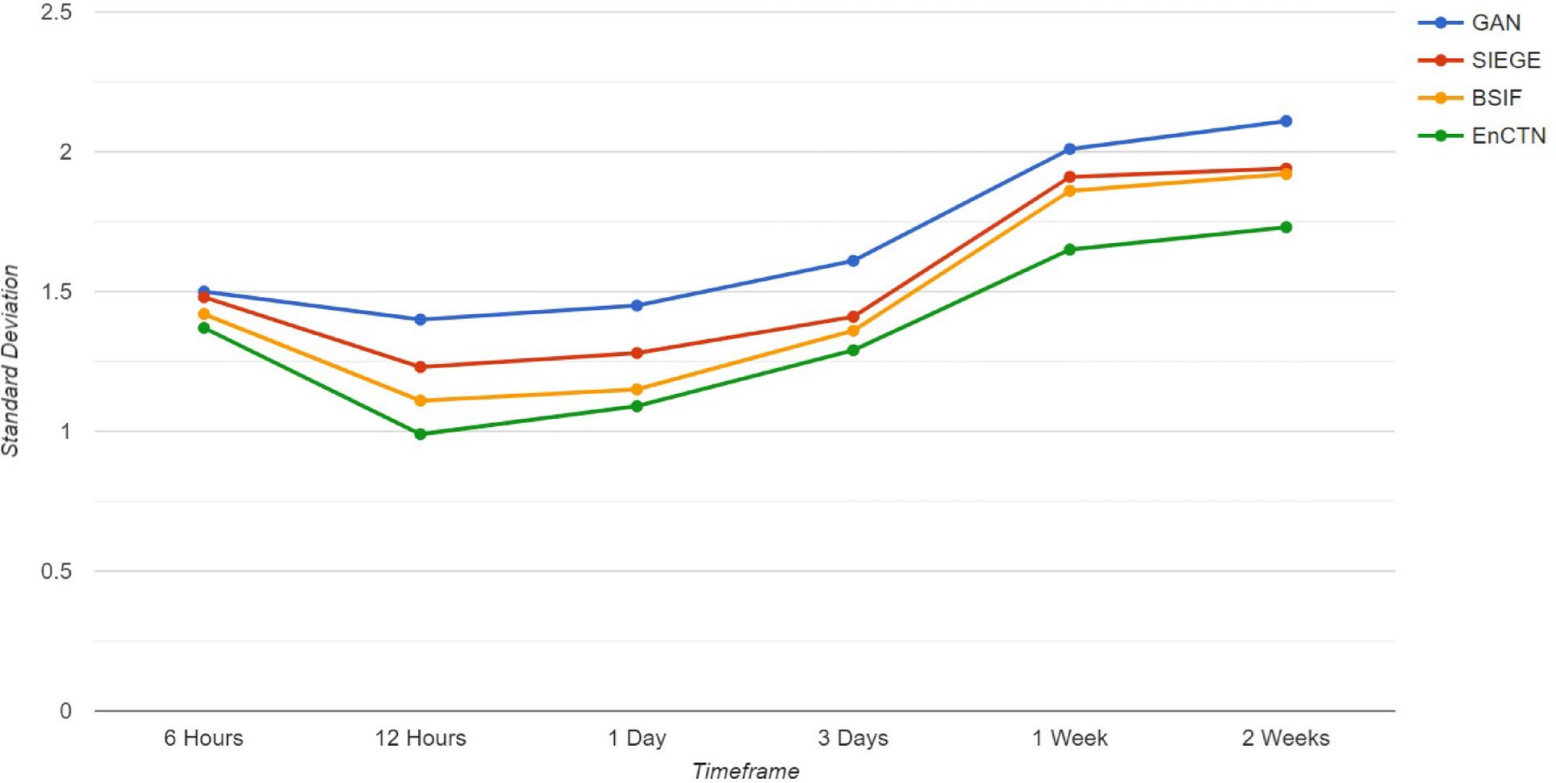
Block time analysis.

Some security ramifications are identified in the process of blockchain transactions, such as 51% attacks, where a miner group controls more than 50% of the network transactions. Replay attacks could involve an attacker retransmitting a transaction. Sybil attacks could create several fake identities to manipulate the network. The block time analysis technique is used to detect 51% attacks by monitoring the time required to generate blocks. Block time refers to the time it takes to create a block within the blockchain framework, specifically the interval between the creation of two adjacent blocks. While tracking the time required for generating every block by computing the mean block time within a specified time, it identifies anomalies that can be discovered through unusual block time patterns and variance in the block time. Gradient Leakage is the main problem in deep learning, and the proposed technique incorporates Batch Normalisation, ReLU Activation, and Residual connections. It can be detected through a monitoring process by reusing the Gradient checkpoint to minimise memory utilisation. The blockchain security is improved by identifying on-chain attacks, such as Anomaly detection, transaction analysis, and decentralised threats with real-time alerts. Gradient Leakage has occurred while sensitive data is revealed through the gradients used to update model parameters during training. Data normalisation is used to prevent the DL gradient leakage. The standard deviation is calculated from the transaction data, like processing time, using the gathered data, and the average value is computed. The variance is calculated for every data point by implementing the squared average difference. With this value, the final standard deviation is calculated. It is very well utilised in blockchain transactions through the Transaction value analysis by identifying unusual patterns that indicate potential attacks and optimising the transaction. Figure 12 demonstrates the Block time analysis given standard deviation, as the proposed EnCTN model has the minimum value compared with the related methodologies.

**Fig. 13:**
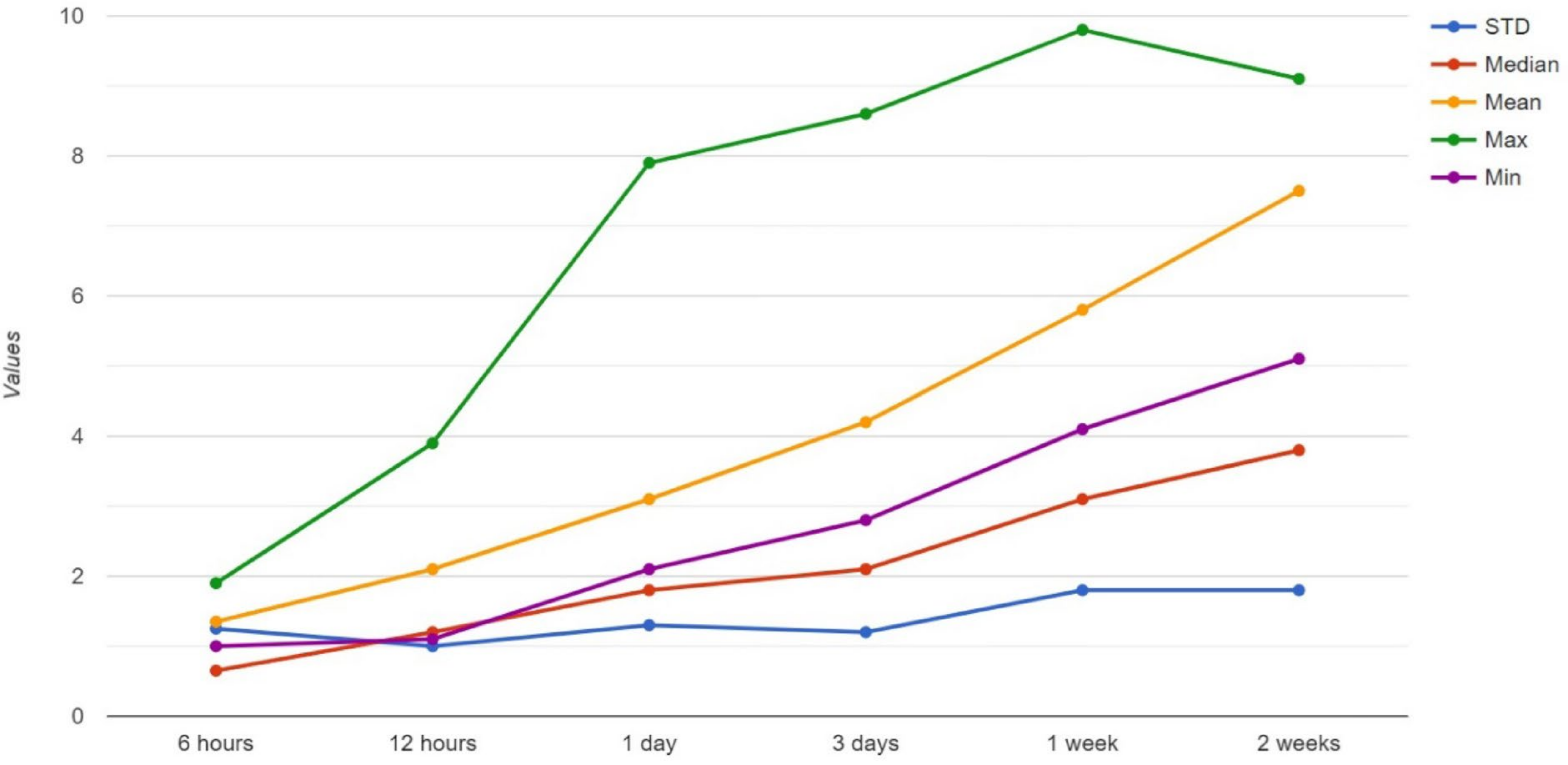
Extracted transaction features at several timestamps.

The execution of the transaction is evaluated through the parameters of Mean value, Standard deviation, Median value, Minimum, and Maximum value. These parameters allow for the recognition of the timestamp of several transactions, enhancing the overall throughput. The transaction ranking is demonstrated in Fig. 13.

**Fig. 14:**
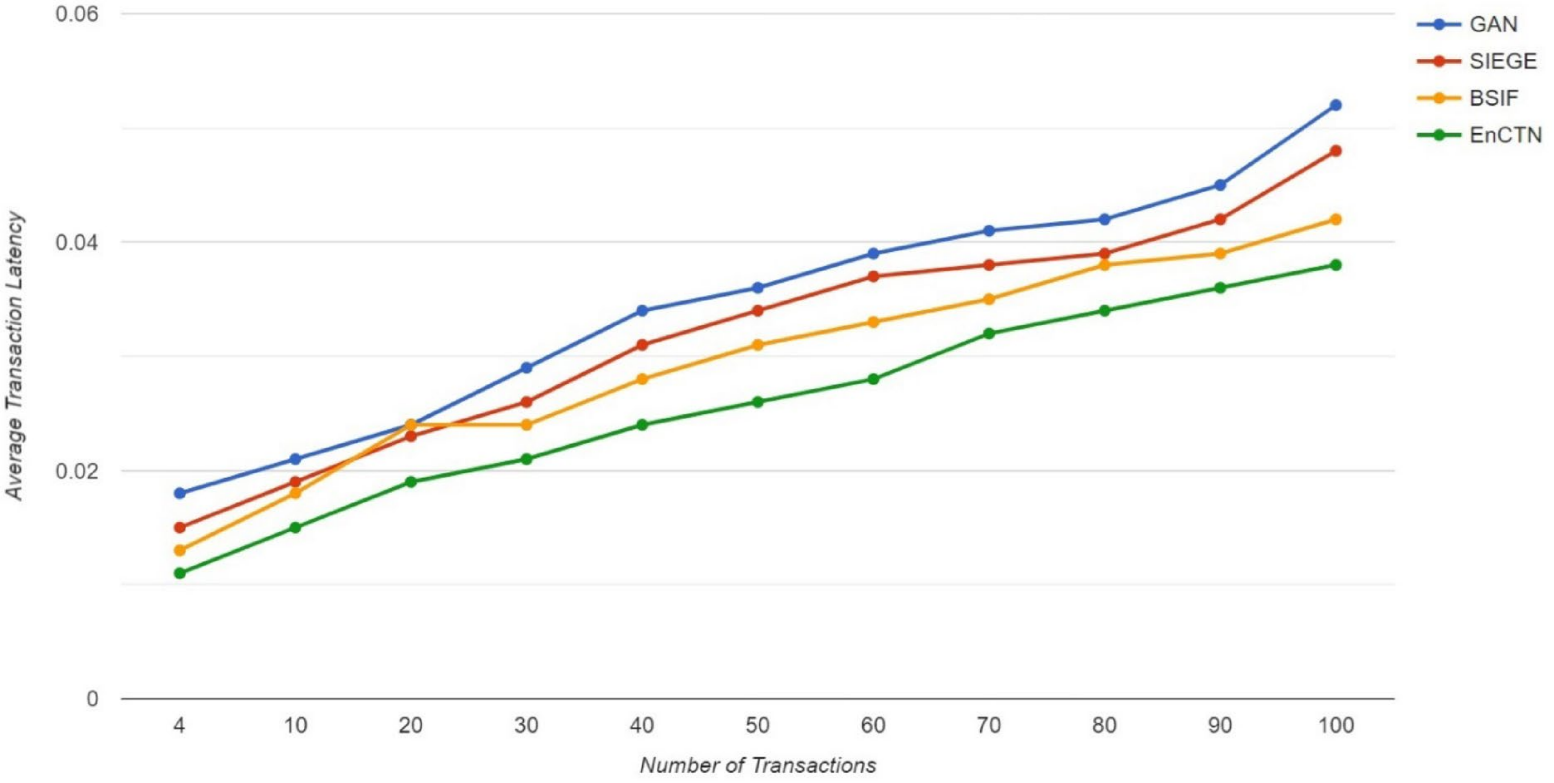
Average transaction latency.

The data faults, like errors and noise, have been removed through the data normalisation process to prevent inconsistency. The error correction code is implemented for deleting transmission errors. The multi-nodes are maintained to ensure data availability to avoid single-point failures. Data faults have been identified through the latency, which demonstrates the time delay within the initial transaction and the confirmation in the blockchain transactions, as it occurs in blockchain creation, transaction verification, and consensus validity. The various factors have affected the latency, such as network congestion, topology, block frequency, complexity, and Fig. 14 demonstrates the Average Transaction Latency, as the proposed technique has the minimum amount of latency compared with the relevant methods.

The on-chain environment in blockchain transaction has the utilization of smart contracts with decentralized framework for executing the transactions in several enhancements in security, visibility of transaction history, the execution of smart contracts to enhance the efficiency by reducing the requirement of intermediate functionality in the distributed network management, the proposed work is constructed to implement the on-chain management for decentralized identity management. Scalability in Ethereum smart contracts demonstrates the ability to handle traffic without affecting security and performance. At the same time, a restricted number of transactions and data storage has enhanced transaction delay, which the proposed technique aims to address with decentralised, smoother transactions. The Ethereum smart contracts have self-demonstrating contracts through pre-defined logic to manage the deployment. The single-point failure has been eliminated on a decentralised framework where tamper-proofness has ensured data storage on the blockchain, and custom tokens are involved in brilliant contract creation.

The performance evaluation from 100 to 1000 TPS illustrates that the proposed model has produced better results in scalability tests for high-demand environments. The related techniques have produced high latency, high power consumption, and the performance degrades in terms of the highest capacity to fail the improved throughput at 1000 TPS. The combined reliability in scalable tests and the minimized energy utilization of the proposed EnCTN make it the optimal choice for processing high-volume transactions and it is illustrated in Table 2.

**Table 2:** Scalability test comparison.

TPS Load	Metric	GAN	SIEGE	BSIF	EnCTN
100 TPS	Latency (ms)	44	59	10	9
	Throughput (TPS)	112	114	122	145
	Power (J)	93	46	33	17
200 TPS	Latency (ms)	92	91	14	10
	Throughput (TPS)	188	191	198	205
	Power (J)	102	88	48	33
300 TPS	Latency (ms)	147	125	15	11
	Throughput (TPS)	208	248	279	312
	Power (J)	195	149	88	45
400 TPS	Latency (ms)	152	140	18	13
	Throughput (TPS)	248	315	351	373
	Power (J)	201	157	105	62
500 TPS	Latency (ms)	188	157	25	18
	Throughput (TPS)	292	358	405	447
	Power (J)	218	167	118	72
600 TPS	Latency (ms)	201	182	35	24
	Throughput (TPS)	300	420	550	600
	Power (J)	225	188	125	88
700 TPS	Latency (ms)	212	182	41	26
	Throughput (TPS)	300	430	635	685
	Power (J)	320	245	133	92
800 TPS	Latency (ms)	232	195	135	94
	Throughput (TPS)	248	345	385	410
	Power (J)	340	285	168	106
900 TPS	Latency (ms)	320	245	133	92
	Throughput (TPS)	285	357	405	423
	Power (J)	348	293	179	118
1000 TPS	Latency (ms)	325	250	140	101
	Throughput (TPS)	293	368	412	440
	Power (J)	362	308	188	127

**Fig. 15:**
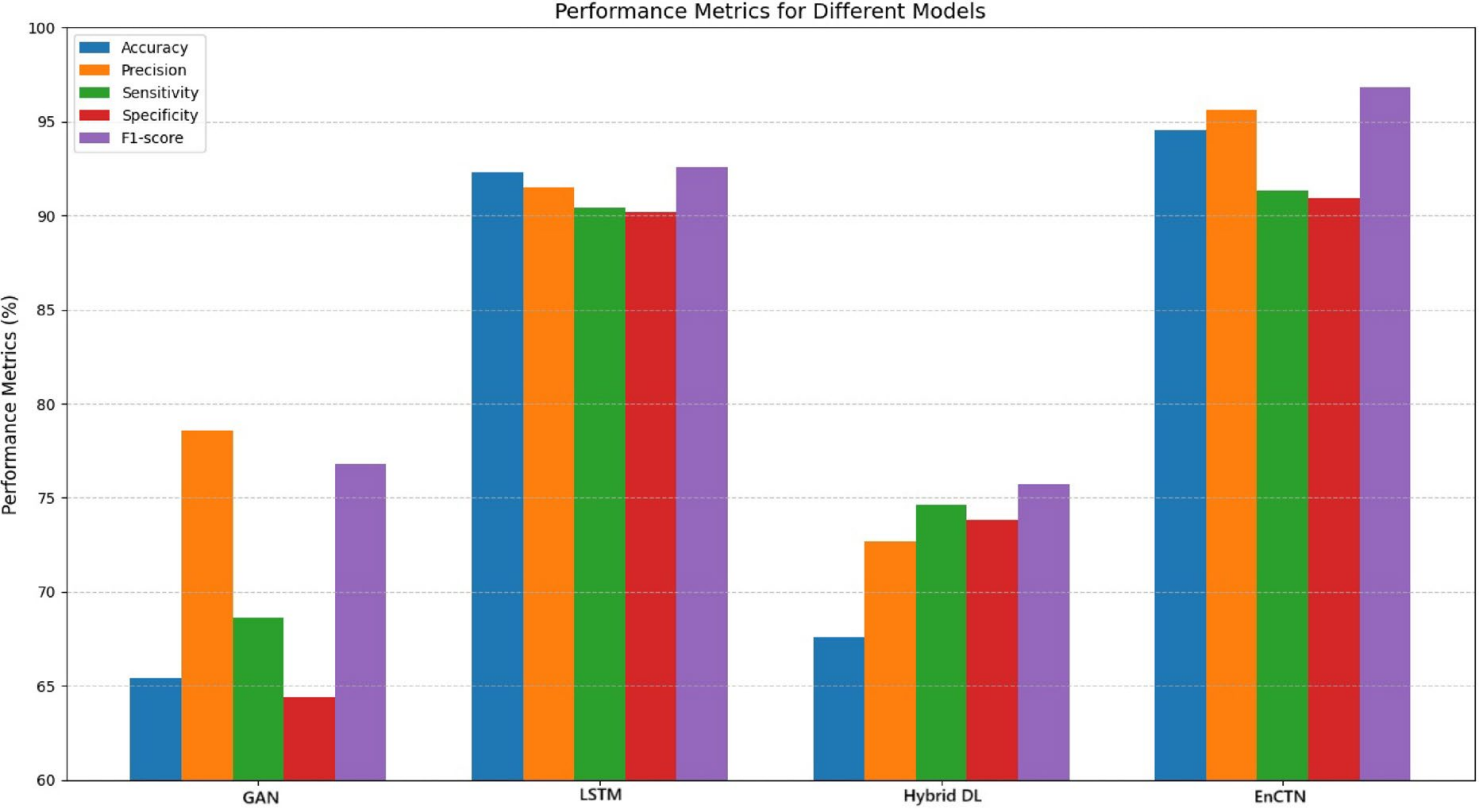
Various performance metrics comparison.

The NSL-KDD dataset^28^ is used to perform the anomaly detection as it has 41 features, like several traffic statistics, service, flag, duration, with the anomalies of Probing, Denial-of-Service, User-to-Root, and Remote-to-Local attacks. The proposed technique is compared with the relevant techniques of GAN^17^, LSTM^19^, and Hybrid DL^27^ on various classification performance metrics. The classification model is evaluated through the performance metrics in view of the classification accuracy as it computes the entire proportion of correct predictions; the issue is the imbalanced dataset. The precision determines the instances of the positive prediction from the actual positive values, while the sensitivity and recall determine the correctly identified model in terms of anomaly detection. Sensitivity demonstrates the measurement of the capability of correctly discovering the negative values as the actual negative instances, correctly measured values. F1-score balances the trade-off between sensitivity and prediction, while the harmonic mean determines the measurement of the compared technique in uneven distribution of the classes, and the comparison is illustrated in the Fig. 15.

The performance evaluation has proved that the proposed technique outperformed the relevant techniques according to the factor-based implication. In contrast, the proposed method utilises the Blockchain-enabled cryptographic primitives for Distributed Deep Learning to manage privacy and computational accessibility. The proposed technique has been framed for the identification of parameters, and the objectivity loss is minimised within the training state. The system has produced the specific output class. Compared with the relevant techniques, the proposed model yields improved results by reducing time complexity with the Deep Learning architecture.

## Conclusion

The transaction security is enhanced through the proposed framework, which is deployed quickly and effectively with the Deep Learning concept, thereby addressing the shortcomings of existing methods. The blockchain-enabled transactions were analysed through the Convolutional Temporal Auto Encoder Technique to identify the outliers. The transaction features are extracted to examine the consensus in the transaction within the specified timestamp. The transactions were executed over 24 h, with random distribution, as the simulation result could prevent the analogous execution in the transactions. Moreover, the integration of the blockchain with the deep learning model could enhance the security of the transaction, and the simulation results have proved the superiority of the proposed framework compared with the relevant techniques. The future work could be expanded to cloud-enabled layers through improved research to provide exact data analysis with low latency. The private blockchain architecture guarantees data privacy through private channels, enhancing access control policies. In contrast, the public blockchain model has been used to solve privacy leakage issues through zero-access control policies.

## Data Availability

The datasets used and/or analysed during the current study available from the corresponding author on reasonable request.
